# A new method for network bioinformatics identifies novel drug targets for mucinous ovarian carcinoma

**DOI:** 10.1093/nargab/lqae096

**Published:** 2024-08-24

**Authors:** Olivia Craig, Samuel Lee, Courtney Pilcher, Rita Saoud, Suad Abdirahman, Carolina Salazar, Nathan Williams, David B Ascher, Robert Vary, Jennii Luu, Karla J Cowley, Susanne Ramm, Mark Xiang Li, Niko Thio, Jason Li, Tim Semple, Kaylene J Simpson, Kylie L Gorringe, Jessica K Holien

**Affiliations:** Peter MacCallum Cancer Centre, 305 Grattan St, Melbourne, VIC 3052, Australia; Sir Peter MacCallum Department of Oncology, The University of Melbourne, Parkville, VIC 3052, Australia; The Faculty of Medicine, Dentistry and Health Science, The University of Melbourne, Carlton, VIC 3010, Australia; St Vincent's Institute of Medical Research, Fitzroy, VIC 3065, Australia; Bioinformatics Division, The Walter and Eliza Hall Institute of Medical Research, Parkville, VIC 3052, Australia; School of Science, STEM College, RMIT University, Bundoora, VIC 3082, Australia; Peter MacCallum Cancer Centre, 305 Grattan St, Melbourne, VIC 3052, Australia; Sir Peter MacCallum Department of Oncology, The University of Melbourne, Parkville, VIC 3052, Australia; Peter MacCallum Cancer Centre, 305 Grattan St, Melbourne, VIC 3052, Australia; Sir Peter MacCallum Department of Oncology, The University of Melbourne, Parkville, VIC 3052, Australia; Peter MacCallum Cancer Centre, 305 Grattan St, Melbourne, VIC 3052, Australia; Sir Peter MacCallum Department of Oncology, The University of Melbourne, Parkville, VIC 3052, Australia; St Vincent's Institute of Medical Research, Fitzroy, VIC 3065, Australia; School of Science, STEM College, RMIT University, Bundoora, VIC 3082, Australia; School of Chemistry and Molecular Biosciences, The University of Queensland, St Lucia, QLD 4067, Australia; Computational Biology and Clinical Informatics, Baker Heart and Diabetes Institute, Melbourne, VIC 3004, Australia; Department of Biochemistry and Pharmacology, The University of Melbourne, Parkville, VIC 3010, Australia; Peter MacCallum Cancer Centre, 305 Grattan St, Melbourne, VIC 3052, Australia; The Victorian Centre for Functional Genomics, Peter MacCallum Cancer Centre, Melbourne, VIC 3052, Australia; Peter MacCallum Cancer Centre, 305 Grattan St, Melbourne, VIC 3052, Australia; The Victorian Centre for Functional Genomics, Peter MacCallum Cancer Centre, Melbourne, VIC 3052, Australia; Peter MacCallum Cancer Centre, 305 Grattan St, Melbourne, VIC 3052, Australia; The Victorian Centre for Functional Genomics, Peter MacCallum Cancer Centre, Melbourne, VIC 3052, Australia; Peter MacCallum Cancer Centre, 305 Grattan St, Melbourne, VIC 3052, Australia; Sir Peter MacCallum Department of Oncology, The University of Melbourne, Parkville, VIC 3052, Australia; The Victorian Centre for Functional Genomics, Peter MacCallum Cancer Centre, Melbourne, VIC 3052, Australia; Peter MacCallum Cancer Centre, 305 Grattan St, Melbourne, VIC 3052, Australia; Sir Peter MacCallum Department of Oncology, The University of Melbourne, Parkville, VIC 3052, Australia; The Victorian Centre for Functional Genomics, Peter MacCallum Cancer Centre, Melbourne, VIC 3052, Australia; Peter MacCallum Cancer Centre, 305 Grattan St, Melbourne, VIC 3052, Australia; Peter MacCallum Cancer Centre, 305 Grattan St, Melbourne, VIC 3052, Australia; Peter MacCallum Cancer Centre, 305 Grattan St, Melbourne, VIC 3052, Australia; Peter MacCallum Cancer Centre, 305 Grattan St, Melbourne, VIC 3052, Australia; Sir Peter MacCallum Department of Oncology, The University of Melbourne, Parkville, VIC 3052, Australia; Department of Biochemistry and Pharmacology, The University of Melbourne, Parkville, VIC 3010, Australia; The Victorian Centre for Functional Genomics, Peter MacCallum Cancer Centre, Melbourne, VIC 3052, Australia; Peter MacCallum Cancer Centre, 305 Grattan St, Melbourne, VIC 3052, Australia; Sir Peter MacCallum Department of Oncology, The University of Melbourne, Parkville, VIC 3052, Australia; The Faculty of Medicine, Dentistry and Health Science, The University of Melbourne, Carlton, VIC 3010, Australia; St Vincent's Institute of Medical Research, Fitzroy, VIC 3065, Australia; School of Science, STEM College, RMIT University, Bundoora, VIC 3082, Australia

## Abstract

Mucinous ovarian carcinoma (MOC) is a subtype of ovarian cancer that is distinct from all other ovarian cancer subtypes and currently has no targeted therapies. To identify novel therapeutic targets, we developed and applied a new method of differential network analysis comparing MOC to benign mucinous tumours (in the absence of a known normal tissue of origin). This method mapped the protein-protein network in MOC and then utilised structural bioinformatics to prioritise the proteins identified as upregulated in the MOC network for their likelihood of being successfully drugged. Using this protein-protein interaction modelling, we identified the strongest 5 candidates, CDK1, CDC20, PRC1, CCNA2 and TRIP13, as structurally tractable to therapeutic targeting by small molecules. siRNA knockdown of these candidates performed in MOC and control normal fibroblast cell lines identified CDK1, CCNA2, PRC1 and CDC20, as potential drug targets in MOC. Three targets (TRIP13, CDC20, CDK1) were validated using known small molecule inhibitors. Our findings demonstrate the utility of our pipeline for identifying new targets and highlight potential new therapeutic options for MOC patients.

## Graphical Abstract

**Graphical Abstract:**
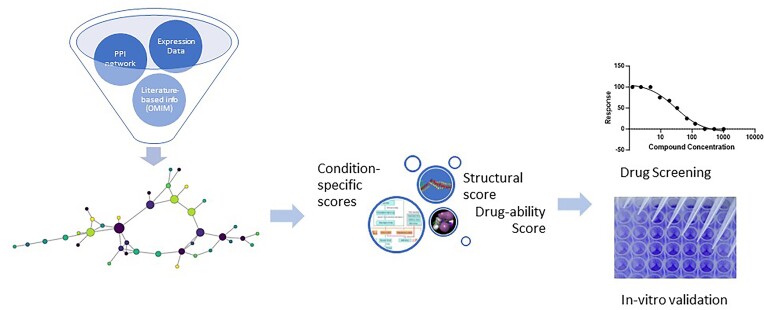

## Introduction

In 2020 alone, over 313 000 new cases of ovarian cancer and approximately 207 000 deaths were reported worldwide (1), making ovarian cancer the 8th highest cause of female cancer mortality (2). Ovarian cancer can be classified into various histological subtypes including; sex cord stromal, germ cell, mixed cell type/miscellaneous and epithelial (3). Epithelial ovarian cancers account for approximately 90–92% of all ovarian malignancies (4), with the most common epithelial histological subtype being high-grade serous ovarian cancer (HGSOC), accounting for approximately 65% of epithelial cases (5); other epithelial histotypes include low-grade serous, endometrioid, clear-cell and mucinous ovarian carcinomas (MOC) (3).

MOC accounts for about 3% of all epithelial ovarian cancers (6) and multiple lines of evidence have found them to be different from ovarian cancers of other histological subtypes. Clinically, mucinous ovarian carcinomas occur at a younger age, with patients more likely to be pre-menopausal, between the ages of 30–50 years old (7), in contrast to other epithelial ovarian cancers that are diagnosed over 60 years of age on average (8). MOC is often diagnosed at an earlier stage (55–75% FIGO Stage I versus HGSOC 5–11% Stage I) (5,9,10) and are often characterised by very large (>10 cm in size), typically unilateral, complex, solid and cystic masses (10,11). This early diagnosis means that patients often do not require chemotherapy and surgical cure is possible (12). However, when diagnosed at an advanced stage, standard of care carboplatin/paclitaxel chemotherapy is ineffective, with a median survival of under 15 months (5). While there is very limited trial data for or against this regimen in this rare subtype, clinically MOC has been reported to respond poorly (13–17). On recurrence or progression after first line therapy the clinical scenario is dire with limited options available, none of which have proven efficacy (12). As a result, advanced disease survival remains poor, and new therapeutic options are desperately needed to improve patient outcomes.

Importantly, MOC show clear differences in gene expression profiles compared with other ovarian cancer histologies (18) and have different genetic profiles (19,20). For example, *KRAS* mutations are present in > 60% of mucinous ovarian carcinomas but are found in <1% of HGSOC. Similarly, there are no *BRCA1/2* mutations in MOC, compared to 25% of HGSOC. This lack of a homologous recombination repair defect is correlated with the lack of response to platinum-based chemotherapy (9). While recent advances in targeting the notoriously ‘undruggable’ KRAS are promising (21), the specific variant being targeted is rare in MOC (G12C present in <5% MOC). The next two most frequent genetic events, *TP53* mutation and *CDKN2A* inactivation, are also challenging targets.

An alternate approach to identify protein drug targets is to map the protein-protein interactions. These approaches are cheap, quick, and can often find important regulators of proteins that would otherwise not been considered in *in vitro* screens. For example, in acute lymphoblastic leukaemia, a *de novo* network model identified two essential genes that were not discovered in the original screen analysis (22). Furthermore, networks-based analyses can demonstrate the dynamic nature of genetic interactions and predict the consistent re-wiring events that occur in response to changing conditions such as chemotherapy (23). Therefore, these *in silico* bioinformatics approaches are a useful way to prioritise potential protein pairs and/or combinations of proteins for functional studies.

For ovarian cancer, these *in silico* techniques have been used to map the protein-protein interactions and network in common forms of ovarian cancer (24,25). Importantly, these methods either did not distinguish the type of ovarian cancer (25) or only utilised a small proportion of MOC samples that were not treated differently than the other types of ovarian cancer (24). Therefore, to date, there has been no MOC-specific protein-protein network published. Furthermore, the ability of these protein-protein interactions to be targeted via small molecule therapeutics i.e. the ‘drug-ability’ was not assessed. Here, we describe a novel bioinformatic pipeline that maps protein-protein interactions with an additional structural bioinformatic filter, leading to novel protein targets which can be readily transferred to a drug discovery program.

## Materials and methods

### RNAseq data

Normalised RNAseq data from MOC and benign mucinous tumours was obtained from https://ega-archive.org/datasets/EGAD00001005190 (20). RNA was extracted from cell lines and organoids using TRIzol (Invitrogen) according to the manufacturer's instructions. RNA sequencing libraries were generated using the same library preparation as the tumours (NEBNext Ultra II Directional Library Prep Kit (New England BioLabs) with ribo-depletion). Sequencing was done using the NextSeq^TM^ 500 System (Illumina) with 75 bp single end reads. RNA sequencing data were aligned to the human genome GRCh37 (hg19) using TopHat2 (26). Sample normalisation was performed using edgeR v 3.42.4 in R v 4.3.1 using RStudio v 2023.03.0 + 386.

### Differential network analysis

Weighted Correlation Network Analysis (WGCNA) (27), a method for gene co-expression network analysis, was used to generate gene-gene networks specific to both malignant (*n* = 31) and benign (*n* = 11) mucinous tumour samples from RNAseq data previously published (20). Benign mucinous tumours are a known precursor to MOC and were used as a control due to the lack of a known cell or tissue of origin. Notably, cases that were borderline morphology (neither carcinoma nor benign) were excluded, as were cases that were not primary MOC (mucinous tumours that metastasised to the ovary). We also excluded cases that were of the sero-mucinous subtype (*n* = 10), as subsequent studies including the most recent WHO classification have shown that these tumours likely have a distinct origin in endometriosis and certainly have a distinct expression profile (28,29). A standard WGCNA pipeline was followed, with a Pearson's correlation adjacency matrix of each dataset raised to the 10th power to soft threshold. Differential network analysis was performed via an implementation of a *diff_i* method (30). The two edge-weighted networks from WGCNA were used as input to generate a single differential interaction network to discover differences in the co-expression networks of the MOC and benign cohorts. Using a manually curated protein-protein interaction dataset gathered through literature and existing PPI databases, we annotated interactions based on the strength of experimental information characterising them, including the experimental methods, the type of interaction and number of publications reporting the interaction. The network was filtered for edges (interactions) that were annotated as ‘physical interaction’ to focus on those interactions that would most plausibly be disrupted via interrupting binding partners. In total this gave us 820 edges involving 659 vertices (unique human proteins).

### Network identification of druggable targets

The weighted network generated via differential network analysis was then used to find druggable MOC specific targets in conjunction with MOC genomic data and structural modelling of protein drug-ability. Somatic point mutations from a cohort of MOC patients with a predicted deleterious consequence were extracted from Cheasley *et al.* (20) and mapped to the nodes of the differential network.

Prize Collecting Steiner Forests (PCSF) (31), a subgraph identification method, which takes both node and edge weights as input was used to identify key subgraphs within the differential network. Node weights were derived from variant score and edge weights from the differential network analysis. Specifically, edge weights were 1 – *diff_i*. The subgraphs that PCSF identifies aim to maximise the number of mutated genes covered while minimising the edge cost to promote sparsity. PCSF was run in randomized edge cost mode where the network edges were sampled with 10% noise 50 times prior to subgraph identification and the union of these runs taken. This step is included to improve the robustness of the final subgraphs to noise.

Separately, protein structures were downloaded from SWISS-MODEL (32,33) and the Protein Data Bank (34) (June 2019). Proteins were assigned a ‘drug-ability score’ based on whether greater than 40% of the protein sequence was able to be structurally modelled. Those which did not have a sufficient structure available (approximately 18.4% of proteins) were assigned a score of 0. These proteins were then assessed using fPocket (35) to determine the presence of a druggable binding site(s). For each protein the highest drug-ability score for all pockets detected on the protein surface was taken to be its overall score. Proteins which could not be modelled or had no detectable druggable site were given a drug-ability score of 0.

To generate a single statistic with which to rank the proteins we used a rank-product scoring metric. Node betweenness centrality in the differential interaction graph, number of occurrences in the PCSF bootstraps, and drug-ability scores were used as input to the rank-product function. For each score, *N* proteins were ranked from 1 – *N* (largest score being rank *N*) meaning that a larger rank product score indicated a more important protein. The minimum rank was used when dealing with ties. The PCSF network was visualised using the ggraph package for graph layouts (36).

### Cell culture

All cell lines were grown in a humidified incubator set at 37°C in 5% CO2. MCAS was grown in alphaMEM supplemented with 10% FBS (Cytiva) and 1% Glutamax (Gibco); JHOM-1 in DMEM:F12 (Gibco) supplemented with 10% FBS, 1% Glutamax (Gibco) and 1% NEAA (Gibco); RMUG-S in Ham's F12 (Gibco) supplemented with 10% FBS; BJ in EMEM (Gibco) with 10% FBS and HFF-1 in DMEM (Gibco) with 15% FBS. HOSE 17.1 was grown in M199:MCDB with 10% FBS. At 70–80% confluence, cells were split in a 1:4 ratio using 0.05–0.25% Trypsin (Gibco). The identity of all cell lines was authenticated using STR similarity profiling.

### siRNA screens

Dharmacon siGENOME™ SMARTpool™ siRNAs targeting each gene candidate were purchased from Horizon Discovery (Dharmacon). siRNA transfection conditions were optimised for each cell line using siGLO™ Red transfection indicator (Dharmacon) in 96-well plate format (optimised conditions detailed in Supplementary Table S1). For each cell line, the optimal DharmaFECT™ Transfection Reagent 1–4 (Dharmacon) was mixed with Opti-MEM™ Reduced Serum Medium (Gibco) according to manufacturer's instructions before complexing for 5 min with 40 nM of either appropriate target siRNA or control: siOTP-NT (On-TargetPLUS non-targeting), siPLK (death), siTOX (death) (Dharmacon). Cells were reverse transfected with the lipid:RNAi complex at an appropriate density such that they would reach ∼80% confluency after 72 h. At 24 h post transfection, a complete media change was performed, before cells were left to grow until the 72 h endpoint. Media was aspirated and cells were fixed with 4% paraformaldehyde, washed and stained with 4′,6-diamidino-2-phenylindole (DAPI) before plates were imaged at 10× across 25 fields per well on the CellInsight CX7 LED High-Content Screening (HCS) Platform (Thermo Fisher Scientific) to generate cell counts per well.

Each plate contained 2–3 replicate wells for each target siRNA and biological replicates were performed twice (BJ, HFF-1, HOSE 17.1) or four times (MOC cell lines). Plates were excluded if the *Z*′ factor was negative (a measure of the differentiation between positive death control siTOX and non-targeting control siOTP) or if the coefficient of variation (CV) of the non-targeting control wells was >20%. BJ and HOSE 17.1 only had one successful biological replicate each and were excluded. RMUG-S had three successful biological replicates and the remaining cell lines had two. Following this quality control measure, target gene knockdown cell counts were normalised to the average of non-targeting control siOTP wells on the plate. For each gene and cell line, outlier wells were identified using as those outside the mean ± 2 × standard deviation of all replicates, which only excluded a single well (RMUG-S TRIP13). Technical replicates were averaged and statistical analysis performed on these values. Each gene and cell line were compared to the normalised controls (i.e. 1) using a one-sample t-test. Cancer cell lines were each compared to HFF-1 using a Wilcoxon test. Statistical tests were controlled for multiple testing correction using the Benjamini–Hochberg method with *P*< 0.05 considered statistically significant. All statistical tests were performed in R Studio v. 2023.09.1 + 494, with R v. 4.3.2.

### Knockdown validation using RT-qPCR

To confirm gene knockdown, RNA was extracted from siRNA transfected cells and corresponding siOTP-NT control transfected wells 24 h post transfection using the RNeasy™ Mini Kit (Qiagen) according to the manufacturer's protocol. The Superscript^TM^ IV VILOTM^TM^ Master Mix (Invitrogen) kit was then used according to the manufacturer's instructions to produce cDNA. cDNA corresponding to each knockdown condition was seeded in a MicroAmp™ Fast Optical 96-Well Reaction Plate (Applied Biosystems) with forward and reverse primers at 200 nM for either the corresponding target gene or *GAPDH* reference gene (primer sequences given in Supplementary Table S2). PowerUp™ SYBR™ Green Master Mix (Applied Biosystems), chosen as the quantifying amplification detection method, was added to each reaction according to the manufacturer's protocol. qPCR was performed using the StepOnePlusTM Real-Time PCR System (Applied Biosystems) with fast cycling parameters, in order to obtain cycle threshold (Ct) values for each amplification condition. A melt-curve dissociation step was also performed to evaluate amplification specificity.

### 2D Small molecule inhibitor screens

RO-3306, Apcin, and DCZ0415 were purchased from MedChemExpress. Cells were seeded in 96-well format at densities as optimised for siRNA and left to grow for 24 h prior to drug treatment. Compounds were diluted in DMSO and dosed in a 10 point dilution series using the D300e Digital Dispenser (Tecan) with 0.05% DMSO normalisation. At 72 h, media was aspirated, and cells were fixed with 4% paraformaldehyde, washed and stained with DAPI. Plates were then imaged at 10× across 25 fields per well on the CellInsight CX7 LED/LZR HCS Platform (Thermo Fisher Scientific) to generate cell counts per well.

### Cell cycle analysis

The raw DAPI images were analysed with CellProfiler v4.1.3 to extract the total DAPI intensity value of each cell. The resulting data were analysed in R using the tidyverse and reshape2 packages. First, a density plot of DAPI intensity values of the negative control cell population was generated. The maximum peak was used to identify the intensity value at the peak of the G_0_/G_1_ phase. This value was then doubled to identify the intensity value at the peak of the G_2_/M phase. The lower and upper intensity thresholds for each cell cycle phase were then set at 35% of the range between the two peaks. Each cell was then classified as below G_0_/G_1_, G_0_/G_1_, S, G_2_/M or above G_2_/M, based on its own total DAPI intensity value, and the percentage cells belonging to each phase calculated for every siRNA knockdown/drug treatment condition.

### 3D small molecule inhibitor screens

MOC organoids were derived from patient tissue following the procedure developed by (37) with minor adaptations. ORG38 was derived from a Stage I infiltrative primary MOC, while ORG64 from a Stage I expansile primary MOC. After culture in Matrigel, organoids were harvested and digested to single cells using Trypl E (Gibco) and seeded in 384-well plates (384-well Flat Clear Bottom Black Corning®) using the Janus G3 Liquid Handler (Perkin Elmer). Following solidification of 10 μl of 80% Matrigel base layer (Corning Phenol Red Free Lot#923-100, diluted with DMEM/F12), 10 μl of cells in a 50% Matrigel/media mixture were seeded to give a final density of 1500 cells/well. After incubating at 37°C in 5% CO_2_ for 20 min to allow solidification of Matrigel, 35 μl of organoid growth media was added to each well using the Biotek EL406 Liquid Handler. Plates were returned the incubator for 72 h to allow organoids to re-form before performing a media change again using the Biotek EL406 and drug dosing using the D300e Digital Dispenser (Tecan). Plates were incubated for another 72 h before another media change and second dosing, then left for a further 48 h before endpoint assays were performed.

Whole-population metabolic activity was assessed using the CellTitre Glo (CTG) Luminescent Cell Viability Assay (Promega). For this, we added 20 μl of undiluted CTG reagent directly onto the 35 μl of media in each well using the automated dispenser (BioTek), sealed the plate, and vigorously shook it on an orbital shaker at room temperature for 20 min. After that, the plates were pulse spun at 1000 rpm, and luminescence intensity was measured at the default gain of 135 on the Cytation5 Cell Imaging Multi-Mode Reader plate reader. Raw CTG data were imported into and processed in R (v4.2.0) using the tidyverse package (v2.0.0).

First, plate heat maps were generated based on raw CTG values at day 8 using the dplyr (v1.0.0) and platetools (v0.1.2) packages to visually confirm consistent patterns to plate layout design, and suspicious wells such as wells with consistently lower values in the outside wells of the plate (potential edge effects) or two adjacent rows having dramatically different values than other rows (potential liquid-handling issue during organoid seeding). Sample well raw CTG values were normalised against the median value of all negative control wells (DMSO).

### 3D cell imaging and Mahalanobis distance

On the final day of drug treatment, nuclei were stained for 60 min at room temperature with Hoechst 33 342 (10 mg/ml) at 1:1000 (Thermo Fisher) using the D300e Digital Dispenser and plates were incubated for 60 min. Brightfield and fluorescent images were taken on live cells (Cytation5 Cell Imaging Multi-Mode Reader (BioTek), 2.5× magnification, one field/well, maximum projection of a stack of three *z*-heights), as described previously (38). Organoid segmentation, based on brightfield images, was performed using CellProfiler software (Broad Institute of MIT and Harvard, version 4.1.3) (39). For subsequent quantification of organoid morphology, including area, radius, and eccentricity, as well as texture and intensity features, we used well-level data, including mean, median, and standard deviation.

To quantitatively assess the morphological changes in the organoids with drug treatment, we employed the Mahalanobis distance, a multivariate distance metric that accounts for the covariance among different features (40). Raw imaging feature data was imported from CellProfiler into the R Studio statistical environment (v1.2.1335) using the tidyverse package (v1.3.0). The image features were then centred and scaled into Z-scores. The Mahalanobis distance was calculated between the feature vectors of organoids treated with vehicle controls (0.2% DMSO) and the drugs of interest. The resulting Mahalanobis distances provided a quantifiable measure of the deviation in organoid morphology with treatment, taking into account the natural variance in the features.

## Results

### Computational identification of potential druggable protein targets for MOC

**Figure 1.**
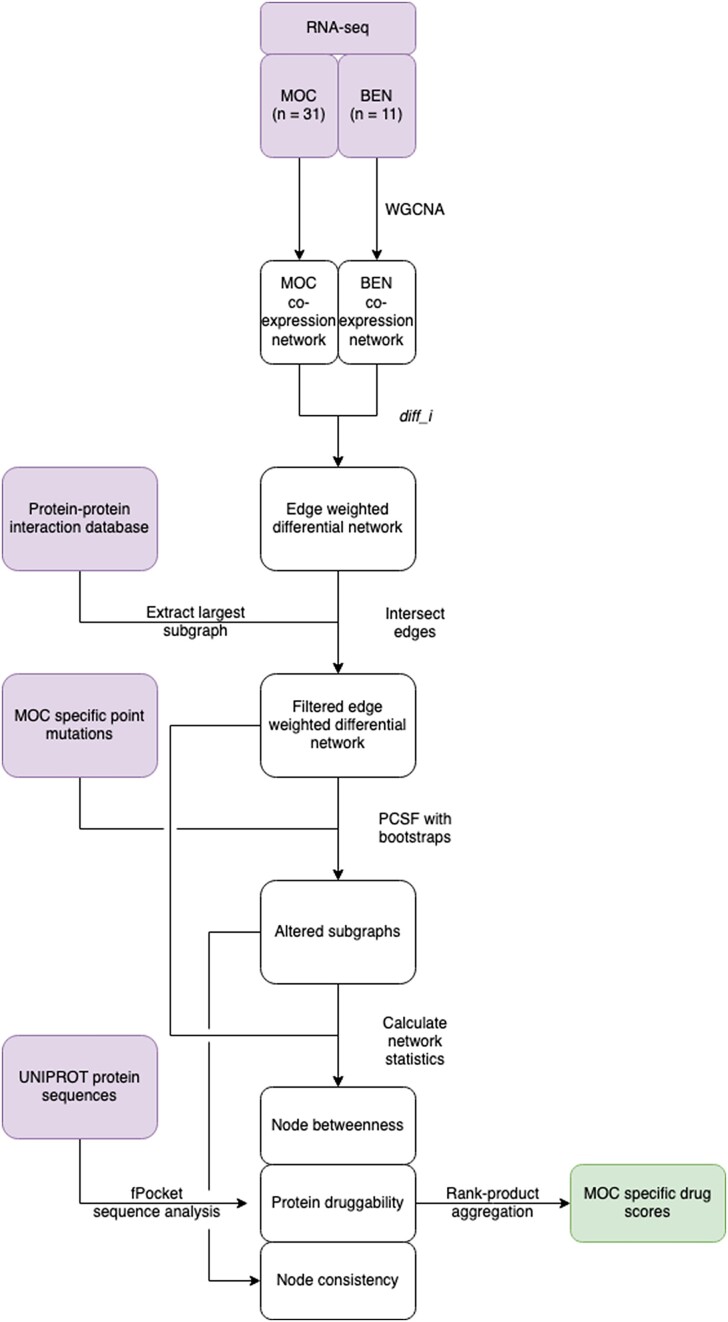
Workflow diagram for the network analysis based discovery of druggable targets. Purple nodes highlight the input data sources with analysis steps connecting intermediates as arrows.

We designed a computational pipeline to identify a set of proteins with both druggable structural characteristics and evidence for gene expression or mutational alteration in a cohort of MOC patients (Figure 1). Briefly, we took gene expression data from a previous study of both MOC and benign mucinous ovarian tumours and created an edge-weighted network of MOC enriched gene co-expression by performing differential network analysis. We refined this network by retaining only those edges with strong evidence for physical interactions by intersecting with a curated PPI network. This network is an amalgamation of multiple online resources (including STRING [https://string-db.org/]) where edges represent multiple types of interactions including literature association, co-expression, and physical interactions. Using these data, the largest connected component of this full PPI graph contained 659 genes with 820 edges between them.

**Figure 2.**
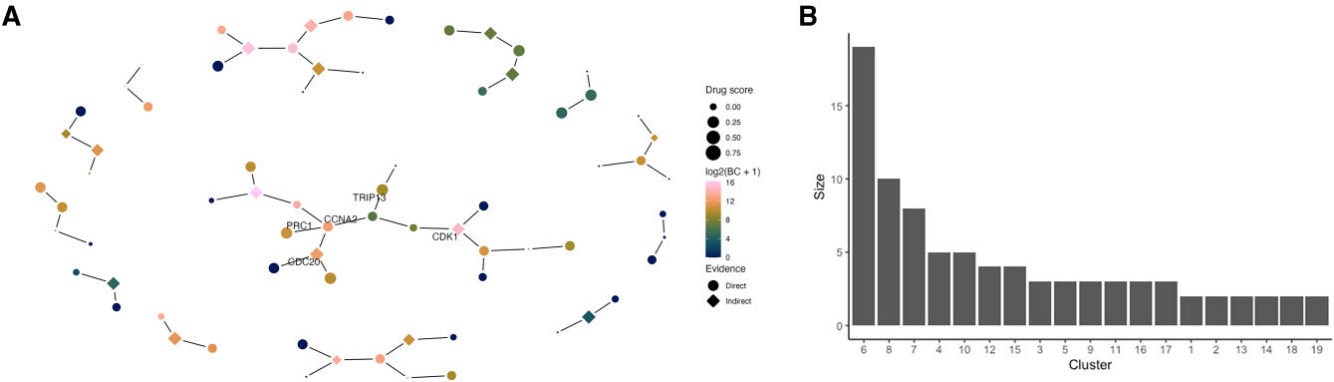
Network analysis outcomes. (**A**) Key subgraphs of the MOC co-expression weighted interaction network identified by PCSF analysis. Nodes (proteins) are coloured by betweenness centrality in the MOC co-expression network and sized by fPocket drug score. Indirect proteins are those without evidence of genomic alteration in the MOC cohort. The five proteins which were selected for validation are labelled. 47 out of the 85 proteins identified by PCSF are shown here. (**B**) Number of proteins per PCSF subgraph. By definition, all subgraphs are minimum spanning trees so have an edge count of 1.

Somatic mutations associated with MOC were then integrated with the differential co-expression network and key subgraphs identified using an implementation of the prize-collecting steiner forests (PCSF) algorithm (Figure 2). The resulting PCSF network had 85 proteins comprising 19 subgraphs; with between 2 and 19 proteins per component (Figure 2B shows the number of proteins per cluster). About 25% (21/85) of these did not have direct evidence of genomic alteration in the MOC cohorts demonstrating the ability of our method to identify novel potential targets by considering protein interactions. Final protein rankings were generated by integrating fPocket derived drug-ability scores, network betweenness of proteins in the input differential network, and the number of PCSF permutations the protein was identified in by calculating a rank product score. Importantly, the information in the drug-ability scores and network betweenness was orthogonal with no significant correlation observed (Pearson's correlation = 0.12, Supplementary Figure S1). Therefore, to balance the dual importance of subnetwork topology and protein drug-ability, we combined the score for both in order to rank proteins as potential drug targets.

The top ranked proteins are given in Table 1, with the full list available in Supplementary Table S3. A literature search was then conducted on these proteins to explore current knowledge of their function and structure. We also assessed whether they had existing small molecule inhibitors available and whether they had previously been linked to cancer (Table 2). This investigation highlighted CCNA2, TRIP13, CDK1, CDC20 and PRC1 as viable targets for our structure-based drug discovery campaign, as they all had a crystallised structure available in the Protein Data Bank, strong evidence of a role in ovarian cancer and known inhibitors (Table 2). Thus, these five candidates were chosen for further biological validation.

**Table 1.** Key proteins identified by our network analysis

Uniprot ID	HGNC symbol	Betweenness	Drug score	Occurrences	Rank product	Type	Cluster	Evidence
Q15831	STK11	48 426	0.95	50	70.97	Steiner	8	Indirect
O43663	PRC1	1618	0.97	50	64.97	Terminal	6	Direct
P06493	CDK1	28 660	0.81	41	63.22	Steiner	6	Indirect
P31749	AKT1	40 964	0.7	50	62.82	Terminal	8	Direct
P36897	TGFBR1	2736	0.81	50	62.16	Terminal	15	Direct
Q13363	CTBP1	1313	0.93	50	61.96	Steiner	8	Indirect
P13497	BMP1	2612	0.79	50	61.21	Steiner	12	Indirect
P20248	CCNA2	5640	0.73	50	60.89	Terminal	6	Direct
Q15645	TRIP13	657	0.98	50	60.57	Terminal	6	Direct
O75398	DEAF1	6916	0.65	50	58.91	Terminal	7	Direct
Q12834	CDC20	4737	0.99	4	34.69	Steiner	6	Indirect

‘Betweenness’ is the betweenness centrality in the differential interaction network and drug score is derived via fPocket. ‘Occurrences’ is the number of bootstraps (out of 50) that PCSF called the protein as enriched.

**Table 2.** Literature analysis of proteins with available crystal structures

Gene	Protein name	Function	Evidence in cancer	Known small molecule inhibitors
CCNA2	Cyclin-A2	Regulator of the cell division cycle by activating cyclin-dependent kinases 2 (CDK2) that participate in the regulation of G1/S phase and G2/M phase, as well as mitotic entry (54).	Upregulated in colorectal (55), breast (56), lung (57) and ovarian cancers (58). Associated with poor outcome.	miR-508-3p (58)
CDC20	Cell division cycle protein 20 homolog	Activates the anaphase-promoting complex/cyclosome (APC/C) to promote the degradation of key regulatory proteins, such as securin and cyclin B1, which are essential for the proper progression through the cell cycle and maintenance of chromosomal stability during mitosis (59,60)	Aberrant expression in pancreatic (61), gastric (62), hepatocellular, lung, oral and bladder cancers (63). High expression in advanced tumour stage of breast (64), colon, endometrium and prostate cancer (65). Upregulated expression in ovarian cancer associated with poorer progression free survival (66)	Apcin (42) TAME and Pro-TAME (67,68)
TRIP13	Pachytene checkpoint protein 2 homolog	Regulates mitotic processes, including spindle assembly checkpoint and DNA repair pathways (69)	Promotes proliferation and invasion of epithelial ovarian cancer cells through modulating the Notch signalling pathway (70). Highly expressed in colorectal (71), lung (72), bladder (73), thyroid (74) and prostate (75) cancers	DCZ0415 (43,44)
CDK1	Cyclin-dependent kinase 1	Control of the eukaryotic cell cycle and regulates the events that occur during mitosis, such as the assembly and disassembly of the mitotic spindle, chromosome condensation, and nuclear envelope breakdown. Also linked to the control of protein synthesis during M phase (76).	Overexpression stimulates proliferation, self-renewal and invasion, seen in several types of cancer such as bladder (77), colon (78), pancreatic (79), breast (80), lung (81), cervical (82) and colorectal (83). Abnormal expression implicated in proliferation and apoptosis of ovarian cancer cells (84)	Dinaciclib (83,85) RO-3306 (41) NSC777201 (86)
PRC1	Protein regulator of cytokinesis 1	Associated with the mitotic spindle midzone during anaphase and localizes to the cell midbody during cytokinesis. PRC1 plays a critical role in regulating the final stages of cell division, where it coordinates cell cycle progression that is controlled by CDKs (87).	Often overexpressed in cancer (88), including prostate (89), gastric (90) bladder (91) and breast (92). Candidate ovarian cancer susceptibility gene (93).	JNJ-7706621 (94,95) Fostamatinib (96)
STK11	Serine/Threonine-protein Kinase, also referred to as liver kinase B1 (LKB1)	Plays an essential role in governing cell polarity and serves as a critical tumour suppressor by regulating the 5′ AMP-activated protein kinase (AMPK) signalling, which governs a wide array of cellular processes including apoptosis, energy metabolism and tumour progression (97,98).	Germline STK11 mutations are associated with lung adenocarcinoma, cervical cancer, and hepatocellular carcinoma (99,100). Additionally, inactivation of STK11 through homozygous deletion has also been observed in lung (101), specifically non-small cell lung carcinoma (102), breast (103) and pancreatic (104) cancers.	CB-839 (100)
DEAF1	Deformed epidermal autoregulatory factor 1 homolog	Encodes zinc finger protein regulates transcription by binding to its own and target gene promoters. DEAF1 is crucial in embryonic development and linked to autosomal dominant cognitive disability when mutated. It activates proenkephalin via protein interaction, inhibiting cell proliferation (G0/G1 phase). Additionally, it is essential for neural tube closure, skeletal patterning, and regulates mammary gland epithelial cell proliferation and side-branching. Finally, it controls peripheral tissue antigen expression in pancreatic lymph nodes (105–110).	Upregulated in breast (111) cancer, whereas overexpression has been seen in several cancer types including liver and gastric cancers, as well as leukemia and oesophageal squamous cell carcinoma (112–117).	AZ505 (118,119), LLY-507 (119,120), A-893 (119,121), BAY-598 (119,122), AZ506 (119), EPZ033294 (119,123,124)
AKT1	RAC-alpha serine/threonine-protein kinase	Involved in various cellular processes including cell survival, growth, proliferation and metabolism (125–130). A central player in various signalling pathways, ATK1 activates the mammalian target of rapamycin, and is also a downstream effector of insulin signalling (131,132).	IncreasedAKT1 activity has been observed in breast, ovarian and prostate cancers (133,134). Overexpression of ATK1 has been observed in colorectal (135), and non-small cell lung cancer (136).	ARQ092,MK-2206, BAY1125976, TAS-117 (137,138)
TGFBR1	Transforming growth factor beta-1 proprotein	A multifunctional cytokine that plays a critical role in the regulation of various cellular processes (139), including cell growth, differentiation (140), immune response (140) and tissue homeostasis (140,141).	Upregulated expression has resulted in poorly differentiated prostate cancer (142,143), high expression of TGFB1 is also associated with mammary epithelial and breast carcinoma (144), pancreatic cancer (140), colorectal cancer (140), lung cancer (140) and metastatic melanoma (140).	AP11014 (145), AP15012 (145), A12009 (145)
CTBP1	C-terminal-binding protein 1	Plays a role in regulating gene expression. It is involved in diverse cellular processes, including regulation of the cell cycle and apoptosis, Embryonic development, transcriptional repression, and cellular metabolism (146,147).	Significant increase in expression found in hepatocellular carcinoma (148) and breast cancer (149).	MTOB(150,151), HIPP derivatives (152,153), Cyclic Peptide CP61 (154), cyclo-SGWTVVRMY (154), NSC95397 (155)
BMP1	Bone morphogenetic protein 1	Plays critical role in bone formation and other various biological processes such as extracellular matrix assembly (156), and tissue development. Some key functions include regulation of growth factor activity (157) and signalling pathways (158), development of connective tissue, wound healing (159) and fibrillin processing (156,159).	Upregulation and high expression have been associated with multiple cancer types including lung cancer (160), osteosarcoma (161), colon cancer (162), renal cancer (163) and gastric cancer (164).	NPL1010(165), NPL3008 (165), K02288 (166).

### siRNA knockdown reduced cell viability in MOC cell lines

siRNA knockdown was chosen as the initial *in vitro* validation method as it is relatively quick to screen multiple targets and its transient mode of action reflects that of a drug. Following determination of the optimal transfection conditions for each cell line, siRNA knockdown of candidate genes was performed for the three currently available MOC cell lines (MCAS, RMUG-S and JHOM-1). Two normal fibroblast lines BJ and HFF-1 were included as well as an ovarian surface epithelial cell line (HOSE 17.1) in order to measure whether any effects seen were cancer cell specific, however only HFF-1 passed quality control.

**Figure 3.**
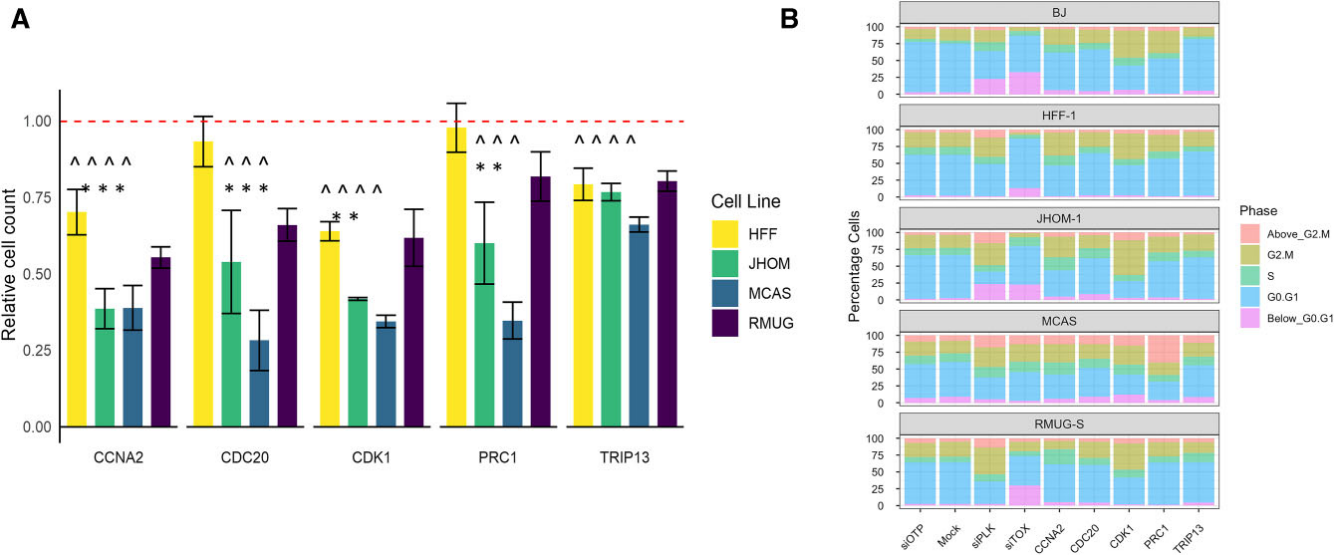
Changes to cellular viability of MOC cell lines following siRNA knockdown of target genes. (**A**) Changes in cell viability for each cell line. Within each cell line, cell counts from each knockdown condition were normalised to the mean siOTP-NT (non-targeting control) cell count of their respective plate and the replicates averaged. Error bars show the standard error of the mean of the biological replicates (*n* = 2 except for RMUG-S which is *n* = 3). Red dashed line shows the level equivalent to no change. ^ indicates statistically significant difference compared to 1 (*P*< 0.05, *t*-test with Benjamini–Hochberg multiple testing correction) * indicates statistically significant difference compared to HFF-1 (*P*< 0.05, Wilcoxon test with Benjamini–Hochberg multiple testing correction) (**B**) Cell cycle stage analysis of siRNA knockdown (technical triplicates). siTOP and Mock are control wells. siPLK and siTOX are expected to kill cells and show strong cell cycle changes.

Knockdowns in MOC cell lines targeting *CDK1*, *CCNA2*, *CDC20, TRIP13* and *PRC1* significantly reduced cell viability compared to the siOTP-NT non-targeting control (Figure 3A, one-sample *t*-test adjusted for multiple testing *P* < 0.05). When compared to the control cell line HFF-1, knockdown of *CCNA2* and *CDC20* was more effective in all MOC cell lines (Wilcoxon test, *P*< 0.05) whereas knockdown of *CDK1* and *PRC1* was only significant at the JHOM-1 and MCAS cell lines (Wilcoxon test, *P*< 0.05).

Given each of our candidate genes have roles within the cell cycle, cell cycle analysis was completed using total intensity of DAPI staining to infer DNA content in knockdown and control cells. Knocking down *CDK1* and *CCNA2* resulted in moderate shifts in the proportion of cells in each phase of the cell cycle. The profiles of *CDC20*, *PRC1* and *TRIP13* knockdowns were similar to those of untreated or non-targeting control treated cells, with the exception of *PRC1* in MCAS which showed a higher proportion of cells above G2/M (Figure 3B).

### Small molecule screening in MOC cell lines mirrored siRNA knock-down results

Three of our candidate genes, *CDK1*, *TRIP13* and *CDC20*, had commercially available small molecule inhibitors. The inhibitor for PRC1 was not commercially available, and we excluded CCNA2 on the basis that the published inhibitors were RNA moieties rather than small molecules. Therefore, we obtained the three known small molecule inhibitors for each target; RO-3306 (CDK1 inhibitor (41)), Apcin (CDC20 inhibitor (42)) and DCZ0415 (TRIP13 inhibitor (43,44)). Compounds were then screened in the same cell lines used for siRNA knockdowns over a 10-point dilution range.

**Figure 4.**
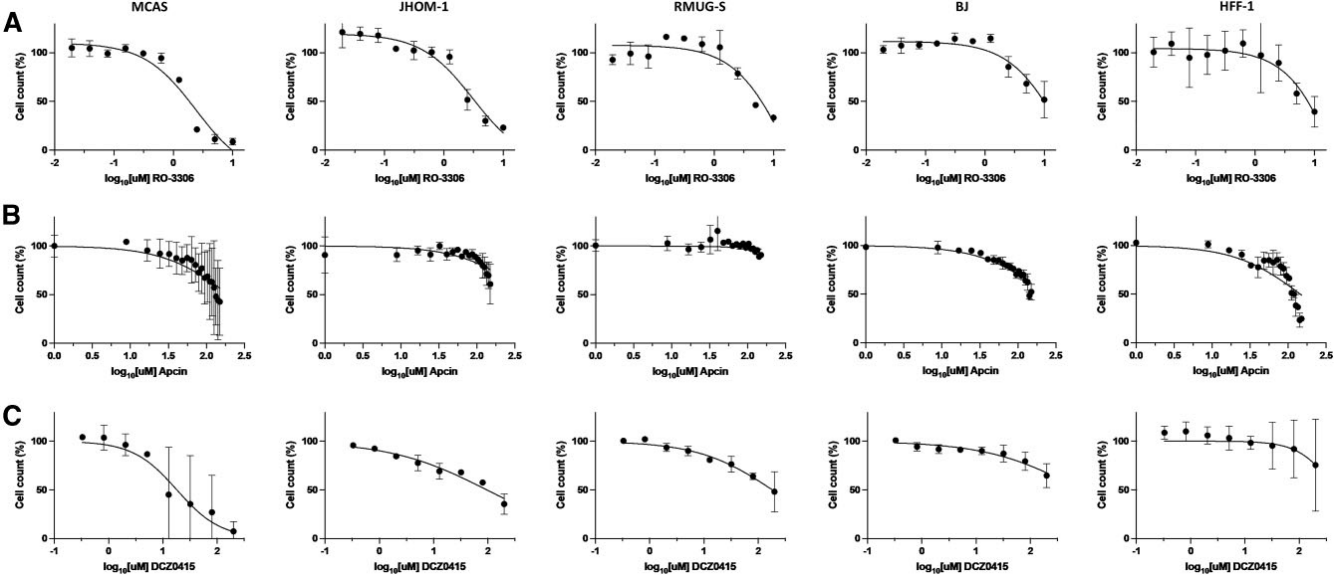
Dose response curves for MOC and fibroblast cell lines treated with small molecule inhibitors of (**A**) CDK1 (RO-3306) (**B**) CDC20 (Apcin) and (**C**) TRIP13 (DCZ0415). Cell count is measured compared to DMSO treated wells. Error bars represent range of the averages of *n* = 3–4 wells from *n* = 2 biological replicates.

**Figure 5.**
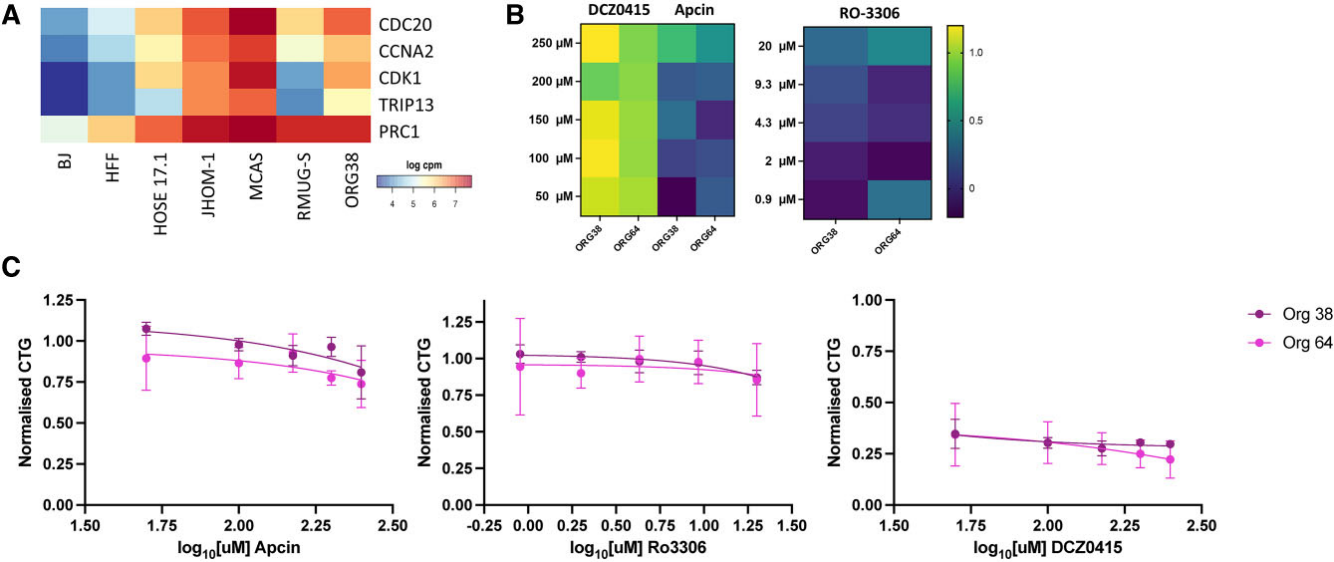
RNAseq and organoid data. (**A**) RNAseq data from organoids (ORG) and cell lines. Not all organoids were used for drug screening but are shown for comparison. CPM – counts per million. (**B**) Results from treating organoids with small molecules against the top targets. Heatmap of log Mahalonobis distance of organoid cultures treated with DCZ0145 (TRIP13), Apcin (CDC20) and RO-3306 (CDK1). A higher value (yellow) is related to a greater distance from control wells. (**C**) Dose response curves for two organoid lines treated with RO-3306, Apcin and DCZ0415. Y-axis is the CellTiterGlo (CTG) of replicate wells (*n* = 5–6 in 3 biological replicates for ORG64, 2 replicate wells for single biological replicate for ORG38) normalised to DMSO control wells.

Small molecule inhibition of CDK1 reduced viability in MOC cell lines JHOM-1 and MCAS, displaying an IC_50_ of 3 and 2.4 μM, respectively for these two cell lines (Figure 4A). In contrast, for the fibroblast cell lines the IC50s were >10 μM, the highest dose tested. The IC50 for RMUG was also greater than 10 μM, however RNA sequencing of MOC cell lines (Figure 5) revealed RMUG-S expressed a lower level of CDK1 than either JHOM-1 (approximately 6-fold higher) or MCAS (approximately 12-fold higher). RT-qPCR quantification of siRNA treatment showed equivalent relative knockdown (>80%), which may be why these cell lines showed similar growth inhibition by this method (Supplementary Figure S2). Taken together, these data show there is a therapeutic window between MOC and normal fibroblast cells for the CDK1 inhibitor RO-3306, suggesting that this small molecule may be a good drug candidate.

For CDC20 inhibitor Apcin we did not see a typical inhibition response curve up to 10 μM, and an expanded dose range showed IC50 values ranging from 196 μM (MCAS) to over 4 mM for RMUG-S (Figure 4B). Apcin reduced viability significantly in MCAS to an extent similar to that seen in either fibroblast line. An effect was also seen to a lesser extent in the JHOM-1 cell line, with RMUG-S only showing minimal reduction in cell number at the highest doses tested. As such, we hypothesise that this may be an example of a drug target that is only relevant to a specific subtype of MOC, given its heterogenous molecular spectrum. CDC20 gene expression was indeed higher in MCAS than JHOM1, with RMUG-S again having the lowest expression of the cell lines (Figure 5). However, the relatively high dose titration used in this assay is unlikely to translate to clinical utility, and thus indicates that an improved CDC20 inhibitor may be necessary for this protein to prove useful as a drug target.

TRIP13 inhibitor DCZ0415 had some effect on the MOC cell lines (IC50s between 17.7 and 174 μM), which was stronger than the effect on fibroblast lines (929 and 465 μM). However, results were somewhat inconsistent between biological repeats, particularly for MCAS (Figure 4C).

### Effect of small molecule inhibitors on MOC organoids

The same three inhibitors were tested in two MOC organoid lines derived from patient tissue, with an expanded upper dose range in order to account for bioavailability and penetration differences when working with 3D matrix-embedded structures. The cell viability results for Apcin were consistent with those of the 2D screens, with a modest reduction in viability seen at high concentrations (Figure 5). A similar curve was produced with RO-3306, demonstrating considerably less toxic effects than those seen in the MOC cell lines (Figure 5C). This lack of effect of RO-3306 in the organoids was confirmed in the M-distance calculations that did not show any changes to the general cell morphology compared to vehicle controls (Figure 5B). Most intriguingly, in direct contrast to the 2D screens, DCZ0415 did produce a significant reduction in cell viability in both organoid lines, even at the lower end of the dose curve, which was confirmed by a widespread change to the organoid morphology based on an increase in Mahalanobis distance (Figure 5B, Supplementary Figure S3).

## Discussion

MOC represents <5% of epithelial ovarian cancer but is a significant contributor to the poor outcome of women diagnosed with ovarian cancer, with a 5-year survival rate of only 25% when diagnosed at advanced stages (45). Recent genomic data identified no readily druggable upregulated protein targets in MOC (18) apart from *ERBB2* amplification in ∼26% of cases. However, *ERBB2* amplification is associated with better overall survival characteristics in MOC (46), meaning that only a small proportion of these amplified cases progress to needing second and later line therapies. Thus, despite the dire need for targeted therapeutics, to date there have been no new identified targets amenable to small molecule drug discovery.

In order to address this, we developed a protein-protein interaction network designed to identify druggable protein pathways upregulated in MOC. Using PCSF, we created these networks and then tailored scoring functions specifically for protein targets that could rapidly be utilised for a small molecule structure-based drug discovery program. In the last 30 years, structure-based drug design has revolutionised the pharmaceutical industry. Structure-based drug discovery methods have been shown to lead to more efficient, specific, and rapid progression to clinical trials from protein target discovery (47). The essential component of structure-based drug design is a protein structure (X-ray, cryo-EM, NMR, or homology model) which contains a defined druggable site (47). Therefore, we designed our pipeline to utilise the plethora of protein structure data available in order to identify protein targets which would be suitable for structure-based drug discovery programs. To do this, we utilised the Swiss-model repository, which contains all types of structures, and added a filter which only kept structures where >40% of amino acids were successfully modelled. Recent advances in machine learning methods for structure determination, such as Alphafold2 (48), have significantly expanded the number of high-quality available structures. Therefore, this would be a valuable addition to future iterations of this pipeline.

We utilised RNAseq data (20) derived purely from MOC patients and benign samples. However, it is well established that levels of gene expression do not always correlate with protein levels. One way to improve this would be by utilising a multi-omics approach incorporating other types of information such as proteomics data (49). Although there are some multi-omics studies described for low-grade serous ovarian cancer (50), this has yet to occur with MOC.

In order to biologically validate our bioinformatic methods, five proteins were knocked down in both MOC cell lines and fibroblasts. Four out of the five targets (PRC1, CCNA2, CDK1 and CDC20) had a significant reduction in growth of MOC cells, with the final target, TRIP13, showed a small difference. All five proteins had clear, established roles in cancer, and three out of the five targets had known selective inhibitors available. Therefore, we purchased the three inhibitors to further explore the role of inhibiting these proteins in both MOC cell lines and 3D organoid models.

RO-3306 is a selective CDK1 inhibitor, developed using structure-based drug development methods (41). It is reported to have direct CDK1 enzyme inhibition (Ki of 35 nM) and a modest cell activity of 9 μM (for complete inhibition of cell cycle at HCT116 and HeLa cancer cell lines) (41). RO-3306 was able to inhibit two out of three MOC cell lines tested and had no significant effect on fibroblasts. Interestingly, at 10 μM, RO-3306 did not have a significant effect on one MOC cell line, RMUG-S. Notably, RNA sequencing showed this cell line expressed >6-fold less CDK1 than the other two cell lines, highlighting the importance of screening targets across multiple molecularly diverse cell lines for heterogenous diseases such as MOC. The effect of this inhibitor in organoids was less striking. It is unknown whether this discrepancy relates to line-specific effects, or the reduced ability for RO-3306 to penetrate 3D structures at the relatively low concentrations used.

Apcin is a modest CDC20 inhibitor with cell-based activities against multiple cancer cell lines ranging from 50 to 100 μM. Against MOC cell lines, Apcin had some activity against MCAS, with little effect on JHOM-1 and RMUG-S. A moderate effect was seen in the fibroblast lines, producing a small therapeutic window between those and MCAS. Taken together, these results indicate targeting CDC20 may only be relevant to particular molecular subtypes of MOC, and even then, an improved inhibitor that is effective at less toxic doses is likely a prerequisite for any future clinical utility in this disease. Notably, recent structure-based methods have developed second generation inhibitors based upon Apcin with 5–10-fold improvement activity for triple negative breast cancer cell lines (51), which may provide a useful avenue for further testing.

Finally, despite not showing a significant effect via siRNA knockdown, we utilised the TRIP13 inhibitor, DCZ0415, against our MOC cell lines. This compound had limited effect on our cell lines, however it did significantly impact the two MOC organoids lines tested. Recently, TRIP13 has been shown to interact with the Wnt pathway in colorectal cancer, where it is significantly more highly expressed in tumours than in adjacent normal tissue (52). We hypothesise that the effect in organoid lines may be related to the dependency of these lines for Wnt supplementation to the media. In ORG38, we have shown that even a 50% reduction in exogenous Wnt3A supplied through the growth media results in loss of organoid growth capacity within three passages (data not shown). In contrast, the cell lines have adapted to standard media and do not require Wnt supplementation. Regardless, the high amount of inhibitor required and lack of efficacy in cell lines suggest this is not going to translate readily into an *in vivo* setting.

Although the pipeline identified novel druggable targets, there is significant room for improvement. Notably, none of the proteins which underwent siRNA knockdown had significant inhibition of the RMUG-S cell line and *TRIP13* knockdown showed no difference in cell growth compared to the fibroblast cell line. As described above, the addition of other forms of data may improve this attrition rate. However, it was promising to see that all proteins identified were druggable with nine out of the top ten all having small molecule modulators identified against them. Furthermore, our siRNA knockdown effect on cell growth was generally mirrored by the effect of small molecule inhibition on cell growth. This shows the potential this approach has, with further refinement, to identify novel protein targets. Furthermore, we only knocked down five of the top targets. Expanding this to more targets, for example AKT1, would potentially highlight this validated cancer target with compounds in clinical trials (53) as a therapeutic target for MOC.

## Conclusion

In this paper, we describe a novel bioinformatic pipeline, which we utilised to create a MOC protein-protein network with drug-ability filters. We identified five candidate genes and performed siRNA knock downs in MOC cell lines and fibroblasts. Further *in vitro* assays utilised known selective small molecule inhibitors against three of these targets in both cell and organoid lines. These results highlight CDK1 as a potential target for MOC patients if improvements can be made since the existing small molecule inhibitor, RO-3306, did not have a significant effect on MOC organoids. Further optimisation may improve this. While we have demonstrated the utility of this pipeline in this rare ovarian cancer histotype, it can readily be applied to any cancer type with gene expression and somatic mutation data available.

## Supplementary Material

lqae096_Supplemental_File

## Data Availability

Unless stated throughout the manuscript, all data is available at: https://zenodo.org/records/12787180.
