# Supplementary material for: A new method for network bioinformatics identifies novel drug targets for mucinous ovarian carcinoma
Source: NAR Genom Bioinform. 2024 Aug 24;6(3):lqae096. doi: 10.1093/nargab/lqae096 (PMC11344246; doi:10.1093/nargab/lqae096)
Supplement: lqae096_Supplemental_File [file lqae096_supplemental_file.docx]

**Supplementary Information**

**
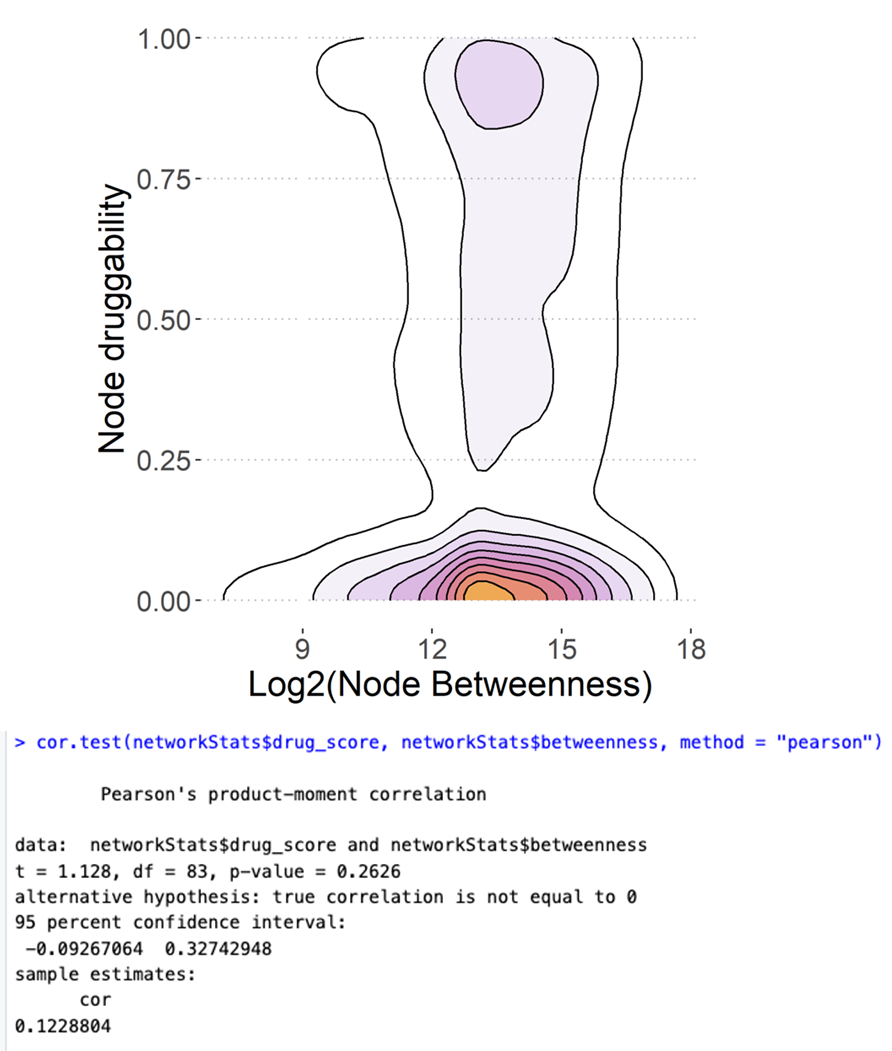
**

**Supplementary Figure 1.** Protein drug-ability score and node betweenness are not correlated. Pearson’s correlation = 0.12

**Supplementary Figure 2.** Relative knockdown of CDK1 across four cell lines. A value of 1 would be no change from siOTP control.


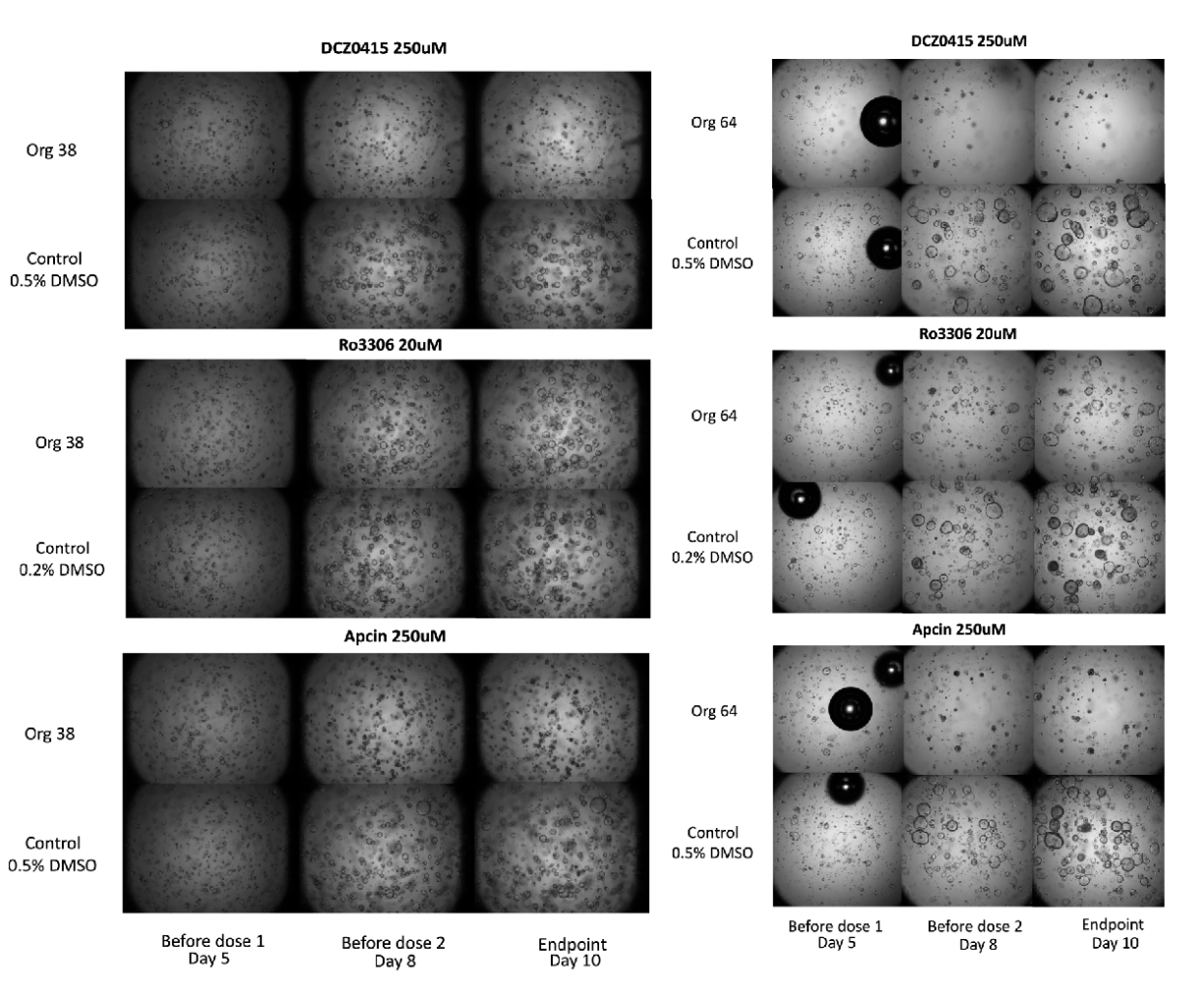


**Supplementary Figure 3.** Brightfield images over time for ORG38 (left) and ORG64 (right) treated with the highest doses of each drug and compared to the DMSO control.

**Supplementary Table 1.** Conditions for siRNA treatment for each cell line

| **Cell line** | **Seeding cell**  **density** | **DharmaFECT ™**  **transfection reagent**  **type** | **DharmaFECT ™ transfection**  **reagent concentration**  **(μL/well)** |
| --- | --- | --- | --- |
| MCAS | 1400 | 1 | 0.2 |
| JHOM-1 | 1200 | 3 | 0.1 |
| RMUG-S | 4000 | 2 | 0.2 |
| HOSE 17-1 | 3000 | 2 | 0.15 |
| BJ | 2000 | 4 | 0.2 |
| HFF-1 | 2000 | 3 | 0.3 |

**Supplementary Table 2.** RT-QPCR oligonucleotide primers

| ***Gene name*** | ***Primer sequences*** | ***Original publication if applicable*** |
| --- | --- | --- |
| *CDK1* | F 5’-ACAGGTCAAGTGGTAGCCATGA  R 5’-ACCTGGAATCCTGCATAAGCA | (47) |
| *TRIP13* | F 5’-GCGTGGTCAATGCTGTCTTG  R 5’- CACGTCGATCTTCTCGGTGA |  |
| *CCNA2* | F 5’-AGTAAACAGCCTGCGTTCACC  R 5’-GAGGGACCAATGGTTTTCTGG | (47) |
| *CDC20* | F 5’-AGATGGACGACATTTGGCCA  R 5’-ATTGGACTGCCAGGGACACC | (47) |
| *GAPDH* | F 5’-GGTGTGAACCATGAGAAG  R 5’-CCACAGTTTCCCGGAG | - |

**Supplementary Table 3.** Top-ranked genes from network analysis

| **Uniprot ID** | **HGNC Symbol** | **Betweenness** | **Drug score** | **Occurences** | **Rank product** | **cluster** | **Evidence** |
| --- | --- | --- | --- | --- | --- | --- | --- |
| Q15831 | STK11 | 48426 | 0.95 | 50 | 357504 | 8 | Indirect |
| O43663 | PRC1 | 1618 | 0.97 | 50 | 274288 | 6 | Direct |
| P06493 | CDK1 | 28660 | 0.81 | 41 | 252720 | 6 | Indirect |
| P31749 | AKT1 | 40964 | 0.7 | 50 | 247968 | 8 | Direct |
| P36897 | TGFBR1 | 2736 | 0.81 | 50 | 240128 | 15 | Direct |
| Q13363 | CTBP1 | 1313 | 0.93 | 50 | 237888 | 8 | Indirect |
| P13497 | BMP1 | 2612 | 0.79 | 50 | 229320 | 12 | Indirect |
| P20248 | CCNA2 | 5640 | 0.73 | 50 | 225792 | 6 | Direct |
| Q15645 | TRIP13 | 657 | 0.98 | 50 | 222264 | 6 | Direct |
| O75398 | DEAF1 | 6916 | 0.65 | 50 | 204400 | 7 | Direct |
| P63104 | YWHAZ | 1312 | 0.75 | 50 | 191632 | 15 | Direct |
| P16220 | CREB1 | 1461 | 0.71 | 50 | 187880 | 7 | Indirect |
| P20700 | LMNB1 | 1975 | 0.67 | 50 | 186368 | 6 | Direct |
| P01730 | CD4 | 37 | 0.96 | 50 | 176792 | 9 | Direct |
| P06748 | NPM1 | 126 | 0.96 | 37 | 168636 | 4 | Direct |
| P29317 | EPHA2 | 9908 | 0.49 | 50 | 168000 | 8 | Direct |
| P08575 | PTPRC | 28 | 0.93 | 50 | 165760 | 9 | Direct |
| Q13526 | PIN1 | 12144 | 0.4 | 50 | 155232 | 7 | Indirect |
| P02462 | COL4A1 | 1310 | 0.59 | 50 | 150024 | 10 | Direct |
| P46777 | RPL5 | 149 | 0.86 | 32 | 141470 | 4 | Indirect |
| Q9NYB9 | ABI2 | 10 | 0.94 | 46 | 139650 | 5 | Indirect |
| P36578 | RPL4 | 125 | 0.78 | 32 | 119970 | 4 | Direct |
| Q13257 | MAD2L1 | 95 | 0.61 | 50 | 115584 | 6 | Direct |
| P20908 | COL5A1 | 659 | 0.42 | 50 | 111888 | 12 | Indirect |
| O43683 | BUB1 | 206 | 0.35 | 50 | 86016 | 6 | Direct |
| Q16543 | CDC37 | 16023 | 0.98 | 5 | 84214 | 8 | Indirect |
| P08670 | VIM | 4563 | 0.59 | 14 | 76176 | 11 | Direct |
| Q9Y5B0 | CTDP1 | 657 | 0.57 | 24 | 66836 | 7 | Direct |
| P16401 | H1-5 | 13122 | 0.38 | 9 | 51480 | 6 | Direct |
| P51617 | IRAK1 | 8436 | 0.68 | 5 | 50986 | 8 | Direct |
| P60228 | EIF3E | 124 | 0.98 | 5 | 46904 | 4 | Indirect |
| Q99661 | KIF2C | 657 | 0.66 | 8 | 44982 | 6 | Direct |
| Q12834 | CDC20 | 4737 | 0.99 | 4 | 41748 | 6 | Indirect |
| O14893 | GEMIN2 | 1 | 0.34 | 29 | 39060 | 2 | Direct |
| Q86X55 | CARM1 | 3265 | 0.98 | 4 | 38080 | 3 | Indirect |
| P33981 | TTK | 993 | 1 | 4 | 32725 | 6 | Direct |
| P02751 | FN1 | 1313 | 0.21 | 14 | 32568 | 10 | Indirect |
| P63279 | UBE2I | 1 | 0.02 | 25 | 23100 | 16 | Indirect |
| P68431 | H3C12 | 79117 | 0.82 | 3 | 22440 | 6 | Indirect |
| O15371 | EIF3D | 46 | 0.53 | 5 | 22386 | 4 | Direct |
| O15169 | AXIN1 | 2622 | 0.58 | 4 | 20790 | 3 | Direct |
| P04908 | H2AC8 | 1051 | 0.77 | 3 | 13664 | 6 | Direct |
| Q04206 | RELA | 11495 | 0.23 | 4 | 13300 | 3 | Direct |
| P25940 | COL5A3 | 0 | 0.75 | 50 | 3360 | 12 | Direct |
| Q04726 | TLE3 | 0 | 0.74 | 49 | 3078 | 7 | Direct |
| P98160 | HSPG2 | 657 | 0 | 50 | 2744 | 12 | Direct |
| Q13330 | MTA1 | 24 | 0.85 | 2 | 2652 | 17 | Indirect |
| Q9Y2U5 | MAP3K2 | 657 | 0 | 48 | 2548 | 15 | Indirect |
| P29466 | CASP1 | 0 | 0.86 | 29 | 2484 | 14 | Direct |
| Q8N201 | INTS1 | 4571 | 0 | 24 | 2170 | 7 | Indirect |
| Q14680 | MELK | 0 | 0.44 | 50 | 2128 | 6 | Direct |
| Q5EG05 | CARD16 | 0 | 0.75 | 29 | 2088 | 14 | Direct |
| P23508 | MCC | 41891 | 0 | 14 | 1992 | 11 | Direct |
| Q99759 | MAP3K3 | 25624 | 0 | 14 | 1920 | 11 | Indirect |
| Q69YH5 | CDCA2 | 0 | 0.62 | 29 | 1764 | 6 | Direct |
| P42336 | PIK3CA | 0 | 0.88 | 12 | 1633 | 8 | Direct |
| P27986 | PIK3R1 | 0 | 0.93 | 10 | 1533 | 13 | Direct |
| O43281 | EFS | 0 | 0.3 | 46 | 1421 | 5 | Direct |
| Q8IY92 | SLX4 | 0 | 0.55 | 25 | 1419 | 16 | Direct |
| O75676 | RPS6KA4 | 0 | 0.23 | 50 | 1344 | 7 | Direct |
| P84103 | SRSF3 | 0 | 0.4 | 29 | 1260 | 2 | Direct |
| Q9H3R5 | CENPH | 1764 | 0 | 8 | 1134 | 6 | Indirect |
| E9PAV3 | NACA | 0 | 0.24 | 30 | 1107 | 1 | Direct |
| Q13490 | BIRC2 | 0 | 0.04 | 48 | 1092 | 15 | Direct |
| Q00613 | HSF1 | 7 | 0.25 | 2 | 1036 | 17 | Direct |
| Q6ZW49 | PAXIP1 | 0 | 0.39 | 15 | 986 | 19 | Direct |
| Q8NEZ4 | KMT2C | 0 | 0.31 | 15 | 870 | 19 | Direct |
| Q9NR12 | PDLIM7 | 0 | 0.24 | 25 | 858 | 16 | Direct |
| P16298 | PPP3CB | 0 | 0.54 | 5 | 546 | 8 | Direct |
| Q8NI77 | KIF18A | 0 | 0.82 | 4 | 469 | 6 | Direct |
| P16403 | H1-2 | 0 | 0.13 | 3 | 88 | 6 | Direct |
| O95785 | WIZ | 0 | 0 | 50 | 56 | 8 | Direct |
| Q02388 | COL7A1 | 0 | 0 | 50 | 56 | 10 | Direct |
| Q14005 | IL16 | 0 | 0 | 50 | 56 | 9 | Direct |
| Q66K74 | MAP1S | 0 | 0 | 50 | 56 | 7 | Direct |
| Q9NPY3 | CD93 | 0 | 0 | 50 | 56 | 10 | Direct |
| Q9P2Y4 | ZNF219 | 0 | 0 | 50 | 56 | 8 | Direct |
| Q96L50 | LRR1 | 0 | 0 | 49 | 54 | 6 | Direct |
| Q9UPY6 | WASF3 | 0 | 0 | 46 | 49 | 5 | Direct |
| Q08AM6 | VAC14 | 0 | 0 | 36 | 45 | 18 | Direct |
| Q8IUZ5 | PHYKPL | 0 | 0 | 36 | 45 | 18 | Direct |
| Q9BWH2 | FUNDC2 | 0 | 0 | 30 | 41 | 1 | Direct |
| Q01167 | FOXK2 | 0 | 0.47 | 2 | 39 | 17 | Direct |
| P12110 | COL6A2 | 0 | 0 | 14 | 24 | 10 | Direct |
| Q9NRJ4 | TULP4 | 0 | 0 | 10 | 21 | 13 | Direct |
